# Exploring the Acceptance and Opportunities of Using a Specific Generative AI Chatbot to Assist Parents in Managing Pediatric Rheumatological Chronic Health Conditions: Mixed Methods Study

**DOI:** 10.2196/70409

**Published:** 2025-07-01

**Authors:** Cheryl W Y Lau, Klaudia Kupiec, Polly Livermore

**Affiliations:** 1 UCL Interaction Centre, Department of Computer Science and Division of Psychology and Language Sciences, University College London, 20 Bedford Way, London, WC1H 0AL, United Kingdom, 44 2074059200; ^2^Rheumatology Department, Great Ormond Street Hospital for Children NHS Foundation Trust, London, United Kingdom; 3 Infection, Immunity, and Inflammation Research and Teaching Department, Great Ormond Street Institute of Child Health, University College London, London, United Kingdom; 4 NIHR Great Ormond Street Hospital Biomedical Research Centre, UCL Great Ormond Street Institute of Child Health, London, United Kingdom

**Keywords:** pediatric health care chatbot, technology acceptance, parental attitudes, children and young people's involvement, chronic disease management, AI hesitancy, chronic health condition, artificial intelligence

## Abstract

**Background:**

Health care chatbots can be used to support patients and their families with everyday decision-making. While there is some research on integrating artificial intelligence into pediatric care, no study has focused on the opportunity of implementing a generative artificial intelligence chatbot for pediatric rheumatology. Pediatric rheumatology conditions require intense family input, which can often leave families struggling to navigate disease flares, pain, fatigue, medication side effects and adherence, and support of their child, often when pediatric rheumatology departments are shut. Understanding how we can support families better, without the need for increased personnel, will have implications for the health care systems.

**Objective:**

The study aimed to explore parental and children and young people’s acceptance of chatbot use in a pediatric context, and understand how a chatbot could be specifically used for managing a child’s chronic health condition.

**Methods:**

This study was a mixed methods design, using both a family workshop and a subsequent questionnaire.

**Results:**

In total, 22 participants contributed to the qualitative design using the world café methodology at a workshop, and 47 participants (36 parents and 11 children and young people) completed quantitative data via a questionnaire. Participants expressed their likelihood of using chatbot technology, including ChatGPT, due to its accessibility. However, participants had significantly greater intention (parents: *P*<.001; children and young people: *P*=.006) to use a specific chatbot over ChatGPT, due to increased trust, credibility, and specificity in design. Children and young people and parents should be distinguished as 2 user groups in chatbot design, reflecting their specific needs in chatbot features and personalization.

**Conclusions:**

Overall, the study reinforced the need for a specialized and trusted chatbot designed with input from health professionals to assist families in managing complex chronic health conditions to support families in between appointments and complement existing face-to-face care. Future research should evaluate users’ engagement with a functional prototype to investigate its usefulness and explore its implementation into families’ everyday lives. Importantly, the current findings have broader implications for the field of pediatric health care, as similarly tailored chatbot interventions could benefit families who are managing other chronic health conditions.

## Introduction

### Background

Generative artificial intelligence (GenAI) tools, such as ChatGPT and health care chatbots, have become increasingly accessible. However, their adoption into health care is still in its infancy. Pediatric rheumatological chronic conditions are autoimmune disorders where the body wrongly attacks itself, as such they have no clear etiology and cure [1]. Juvenile idiopathic arthritis is the most common rheumatological condition affecting one in every thousand children and young people, causing painful joint inflammation and damage often into adulthood [2,3]. Trial and error of treatments are often needed to manage symptoms and hopefully send children and young people into remission, where the disease becomes inactive. Parents need to take charge of managing their child’s health, especially when health professionals are unavailable [4]. Thus, managing a chronic condition can cause a psychological burden to children and young people and their families.

Health professionals often use medical jargon, while parents favor more neutral and simpler terminology for their children [5]. This widens a gap in effective family-clinician communication, which could manifest in medical nonadherence and impact overall treatment efficiency [6]. As parents need to interpret complex information to an appropriate comprehension level for their children, their emotional burden also increases [7-10].

Emerging research on chatbots has demonstrated their opportunities and limitations in health care applications. ChatGPT is a general large language model (LLM) that has been found to be feasible in generating detailed and empathetic responses to patients’ health inquiries [11]. Yet, it is prone to fabricate academic sources, a phenomenon known as “hallucination” [12]. ChatGPT also requires clear input from users to generate more accurate responses, than using ambiguous prompts [13].

Existing health care chatbots have shown promise in improving patient outcomes through integrated care [12,14]. Seeking health information web-based health information could be appealing due to its convenience, availability, and privacy when health professionals are unavailable [15]. Compared to general internet resources, chatbot use could reduce engagement with inaccurate and often overwhelming amounts of information on the internet [16]. However, maintaining clinician-patient communication remains important to prevent self-diagnosis [17]. Therefore, there is a need for a trusted and accessible resource to support families’ decision-making, alongside health care providers.

It is crucial to understand users’ adoption intention to ensure successful embedment of artificial intelligence (AI) intervention into their everyday lives [18,19]. Based on Ajzen’s [20] theory of planned behavior, research on chatbot acceptance in health care [21,22] has identified key factors influencing adoption intention. The theory posits that an individual’s behavior is determined by their behavioral intention, which is influenced by personal attitudes, subjective norms, and perceived behavioral control. Personal attitudes are suggested to be the most influential factor of behavioral intention as attitudes reflect individuals’ own beliefs and schemata [20,23]. For example, Nadarzynski et al [21] found that perceived usefulness, perceived ease-to-use, and curiosity, predicted acceptance of chatbots, though lack of trust and humanness remain concerns. In pediatric care, parental attitudes toward general AI use reveal safety and ethical concerns [24,25].

A limitation of existing studies is that findings might not be generalizable to children with diverse medical backgrounds. For instance, only 24 out of 804 (3%) parents had experience with child hospitalization in Sisk et al [26]. Haley et al [27] found that parents of a child who had been hospitalized significantly prioritized AI accuracy, than those who had not. These families might have unique experiences with medical care, increasing barriers to adopting a novel intervention.

Existing research has also neglected children and young people’s involvement as they often considered parental attitudes. Involving users in the development of AI health technology could increase transparency and trust while preventing dissatisfaction and disengagement [28,29].

### Research Aims

The ongoing “IMPACT” (Interventions to Improve Mental Health Support in Families With Children and Young People With Chronic Rheumatological Conditions) project [30] aims to design, develop, and test a chatbot intervention to support families between appointments for pediatric rheumatological conditions. However, as part of this work, an opportunity arose to gain an understanding of parental and child thinking surrounding AI in general in health care. We, therefore, aimed to explore the attitudes and technology acceptance perspectives of parents and their children and young people. The overall aim is not for a chatbot to replace existing traditional care, but to enhance the support of families between appointments.

This study consists of 2 methodologies. The first piece of work presents data from a family co-design workshop, where families’ opinions on using a chatbot in a pediatric health care context were gathered. Subsequently, a questionnaire was conducted to follow up on the findings identified at the workshop. This mixed methods design allows further probing of the data from the workshop and thus aids a richer insight into perspectives of AI in health care. This study presents both (1) the workshop and (2) the questionnaire, split into separate methods and results, with a combined discussion.

This study aims to address the following research questions (RQs): (RQ1) What are parental and children and young people’s general attitudes and behavioral intentions toward chatbot use, such as ChatGPT? (RQ2) What are parental and children and young people’s attitudes and behavioral intentions toward the potential use of a chatbot specifically designed for pediatric rheumatology and do they differ when considering ChatGPT? (RQ3) What are the opportunities and concerns parents and children and young people might have toward chatbot features and content?

## Methods

### Study Design

The methods section will first present information relevant to the workshop, followed by the questionnaire. This study used a mixed methods design. The main aim was to use qualitative data to understand the acceptance and use of a rheumatology-specific chatbot for parents. Quantitative data have been added to briefly outline the current use of GenAI or similar, with the main focus investigating the attitudes and intentions of using AI to inform health care management. The main target audience was parents, however, as their children also attended the family workshop, we felt it would be an opportunity to seek their perspectives.

### Ethical Considerations

Ethical approval was granted by the Research Ethics Committee at National Health Service (NHS) Health Research Authority, Leeds West Research Ethics Committee, and HRA and Health and Care Research Wales (IRAS 329476). The research project has been reviewed and approved locally at Great Ormond Street Hospital for Children NHS Foundation Trust for capacity and capability. The “IMPACT” study officially opened at Great Ormond Street Hospital for Children on November 10, 2023 (study: 23IR07). Informed consent was sought from all participants. Secondary analysis utilising existing data from the IMPACT study was obtained with primary consent for participating in the project. Quantitative data was fully anonymised, whilst qualitative data was deidentified as outlined in the participant information sheets and consent forms. Participation was voluntary, and no compensation was received.

### Family Workshop (May 2024, London)

#### Design

The workshop used the world café forum method, a qualitative participatory method used in health research [31-34]. In groups, participants rotate around tables to take part in different discussions. The host of each table remains to facilitate the cross-pollination of ideas from previous groups [33]. Participants are encouraged to express themselves creatively in texts or drawings [32].

The corresponding author acted as a table host to guide discussions. A young adult in remission from juvenile idiopathic arthritis and their parent acted as facilitators to maximize participant comfort and engagement, as they shared similar experiences. Another researcher acted as a notetaker. As part of the larger study, there were 7 other tables with different research focuses.

At our table, participants familiarized themselves with 3 sets of questions and ChatGPT responses regarding pediatric rheumatological conditions in a concept testing activity (Multimedia Appendix 1). These 2 conditions were used in relation to participants’ personal experiences. ChatGPT was prompted to generate responses appropriate for either a 6-year-old child or a 13-year-old teenager. Participants were then asked to discuss their likes, critiques, and opportunities regarding these responses.

#### Participants

Families living with a pediatric rheumatological condition requested to attend the workshop after receiving a flyer from their local health care team across the United Kingdom as they attended clinical appointments or via social media advertisements from relevant charities. There were no restrictions on who could attend and whole families were encouraged to attend. To protect families’ anonymity, limited demographic and identifiable information was collected. Inclusion criteria were that families needed to have experience with a pediatric rheumatology condition and be able to speak English. The participants had received rheumatology care from different parts of the United Kingdom. The main target group for this study was parents and carers, as the chatbot created as part of the IMPACT study is targeting parents and carers in the first instance. However, as children and young people also attended the workshop with their parents, their views were also explored to understand the differences and similarities between caregivers and children and young people.

#### Materials

As the world café method collects creative qualitative output, participants were provided with sticky notepads, colored pens, and paper to share their ideas. Printed handouts of ChatGPT responses were also provided. A computer was used to host a live interactive demonstration of ChatGPT.

#### Procedure

Participants were divided into 8 groups that rotated among 8 tables every 20 minutes, with 3 breaks provided to avoid fatigue. Prior to the main activity, the host, facilitators, and participants introduced themselves to one another.

To enable conversations to occur freely, audio recording was not conducted at any table at the workshop. However, detailed notes were taken by 2 notetakers, who then cross-compared their notes to ensure objectivity [35]. Hand-written notes by the participants were also collected. At our station, we conducted a live demonstration using ChatGPT to ease participants into the discussion. Then, participants were asked a set of structured questions and probes, 5 minutes after reading the ChatGPT responses. At the end, all participants and researchers gathered to have a debrief.

Other tables had similar setups. Detailed compiled notes were shared among the researchers electronically postworkshop to be used as secondary data if relevant. One of the other tables explored participants’ consensus on chatbot features in a brainstorming activity, which generated relevant findings to answer RQ3. The findings from the brainstorming activity were summarized by notetakers and therefore no transcripts are available. This strategy helped enrich the dataset and allowed cross-comparisons between data compiled by different researchers, increasing interrater reliability.

#### Analytical Approach

A qualitative descriptive method was followed to stay close “to the surface of words and events” on a semantic level [36]. Trends were noted as the groups progressed leading to data saturation, but with specific attention to ensuring all participants were encouraged to share their perspectives, Since notes taken from the world café forums were already interpreted to some extent, this approach could mitigate further interpretative bias. The “IMPACT” Chief Investigator (PL), who is a senior pediatric rheumatology nurse with qualitative expertise, and Research Facilitator (KK) oversaw the data process and analysis.

For the concept testing activity, a deductive approach was appropriate as the questions were designed according to the predefined categories of “likes,” “critiques,” and “opportunities.”

### Questionnaire

#### Design

The questionnaire design was informed by our workshop findings and a pilot study (n=22) was conducted to test the comprehensibility and relevance of questions. A nonstandardized questionnaire was devised for the purpose of this study to allow for bespoke questions to build upon workshop findings. The potential issues of language barrier, AI literacy, and children’s comprehension level were considered. It was decided that the wording of questions should be kept simple and the number of question items on each construct should be reduced.

Using pilot feedback from the workshop, iterations were made. Initially, the term “ChatGPT” was used instead of “generative AI” or “chatbot” in the questions to provide clarity to those with less knowledge in GenAI. However, it might have measured attitudes specifically toward ChatGPT. For example, a parent said they would not use ChatGPT because “it doesn’t give source references for its reply.” While fabrication might be observed in ChatGPT, a specific chatbot could eliminate this issue. Therefore, questions were rephrased to measure and compare attitudes toward ChatGPT or similar GenAI and a rheumatological-specific chatbot. The final questionnaire was distributed electronically to ensure completion.

#### Participants

Participants were recruited separately from the workshop. Using a purposive sampling method, families who had already expressed interest in the “IMPACT” study were emailed the questionnaire link. Parents were the point of contact and were asked to share the child questionnaire link with their child if they were aged 8 years and older and wanted to take part. We asked that each family be represented by one parent only, but their children and young people may have also completed a questionnaire. This sample might have overlapped with the sample of the workshop and pilot study due to the anonymity provided through the questionnaire, the numbers of these are therefore unknown.

#### Materials

Two versions of the questionnaire were designed, one for parents and one for children and young people, consisting of 13 and 11 questions, respectively. Participants were asked for their age, current internet health information–seeking behavior, and their current use of ChatGPT or similar GenAI. Then, participants were introduced to ChatGPT. Next, they were asked about their behavioral intention of using ChatGPT for seeking health information. This was followed by a short answer question where participants were asked to explain their rating. Participants were then presented with a scenario of a health care chatbot specifically designed by health professionals for pediatric rheumatological conditions. They were asked to rate their intention toward using this specific chatbot and explain whether their attitudes differed regarding the general and specific chatbot. Additionally, parents were asked whether their behavioral intention to use a chatbot for health care differed when it was for their child’s health or their own health.

#### Procedure

The questionnaire was reviewed by the “IMPACT” Chief Investigator and Research Facilitator for iterations. The questionnaire was distributed through REDCap (Research Electronic Data Capture; Vanderbilt University) and sent out via email on June 21, 2024, to 60 families. A reminder was sent on June 25, 2024, and access to the questionnaire was closed on July 1, 2024.

#### Analytical Approach

Using Likert rating and open-ended questions, the questionnaire generated both quantitative and qualitative data. To investigate whether there is a within-group attitudinal difference in using ChatGPT and a rheumatologically specific chatbot for health, a post hoc 2-tailed Wilcoxon signed rank test was conducted. As the data were not normally distributed, the nonparametric design was used to interpret the data. Reliability measures were not used due to the minimal questionnaire design, considering sample characteristics. SPSS Statistics (version 29; IBM Corp) was used for quantitative analysis.

Braun and Clarke’s [37,38] and Bryne’s [39] 6 phases of reflexive thematic analysis were used to interpret the qualitative data. The steps followed included (1) familiarization with the data where participants’ responses were read through to understand the overall tone and content; (2) ascribing primary codes; (3) secondary inductive coding; (4) clustering codes together to identify common ideas, factors, and findings; (5) transforming clusters into relevant and understandable themes; and (6) creating the report [37-39]. The entire analytical process and any discrepancies were regularly discussed with the “IMPACT” team (PL and KK). An audit trail and reflective data were kept at every step. An inductive thematic approach was used to analyze open-text responses to avoid overlooking unexpected themes [37]. Data were analyzed at a semantic level, staying true to participants’ perspectives, which is appropriate for understanding individual stories and contexts in health research [40].

## Results

The results section will first present the family workshop findings, followed by findings from the questionnaire.

### Workshop Findings

Two tables at the co-design workshop explored possible chatbot features and the opportunity of using a chatbot in pediatric rheumatological care, respectively. In total, there were 8 groups of mixed participants which included children and young people (n=9; age range 5‐26 y; mean age 13.3, SD 6.3 y) and parents (n=13). Each group consisted of 2‐5 participants.

### Chatbot Features

#### Personalization

Participants felt that the chatbot needs to evolve with them. For instance, families felt that simple language should be used at the beginning of diagnosis. However, explanations should become more advanced in the subsequent years as families become more familiar and do not want to feel patronized. An opportunity proposed by one participant was the idea of a “science-ometer” slider for users to dictate how scientific they wish the explanation to be. Overall, participants thought that the chatbot should be tailored to the individual patient’s age, personality, and neurodiversity.

#### Tone

Participants thought that the specific chatbot should have a friendly, reassuring, and trustworthy tone. However, they attributed most of the trustworthiness to the endorsement by the NHS behind the design of the chatbot. Children and young people thought that the chatbot should not feel medical at all but like a peer. However, parents wanted to talk to a mentor figure, whether that is a parent with more experience or a health professional.

#### Anthropomorphism

The younger participants wanted to pick an animal avatar that “felt right” for them, such as a dolphin. This feature was less important for older participants and parents, who prioritized the content. They also expressed that the chatbot name or avatar would not affect their intended use. However, they also proposed other symbols to represent the chatbot, such as a torch or volcano.

#### Concept Testing

Conducting concept testing is advantageous in the early development of an intervention to reduce the risk of users not liking the concept, leading to limited adoption [41]. Table 1 provides a summary of users’ likes, critiques, and posed opportunities.

**Table 1.** Summary of concept testing findings.

Categories	Comments
Likes	Visual Imagery aids explanation and understanding. Storytelling narrative is relatable. The more technical version actually explains how the illness works well.
Critiques	Children of similar ages do not always think in the same way, since they are exposed to different things. Unless age is put into the prompt, users are shown information that is too confusing. The analogy used in the 13-year-old explanation attempted to sound more technical but still used the castle analogy that was used in the explanation for a 6-year-old. It was confusing and inappropriate.
Opportunities	Doctors’ and experts’ input is required for the chatbot to be reliable. Doctors should be able to see a record of questions asked by the families to aid communication. The child should be considered as a user for autonomy.

### Questionnaire Findings

#### Overview

A total of 47 participants were recruited, including 36 parents and 11 children and young people (see Table 2 for demographic information). Responses to closed questions are summarized in descriptive statistics first, followed by statistical analyses. Qualitative findings are supported by the use of participant quotes (“P” denotes parent and “CYP” denotes child or young person quotes). Subsequently, qualitative analysis of responses to open-ended questions is discussed.

**Table 2.** Demographic information of parent and children and young people participants^a^.

Characteristics	Parents (n=36)	Children and young people (n=11)
Age (years)		
Age of child or self, mean (SD)	11.1 (4.0)	14.8 (5.9)
Range	4‐19	9‐29
Time since diagnosis of child or self, n (%)		
Less than 6 months ago	0 (0)	0 (0)
6‐12 months ago	3 (8)	1 (9)
Over 1 year ago	6 (16)	0 (0)
Over 2 years	27 (73)	10 (91)
Diagnosis, n (%)		
JIA^b^	14 (39)	4 (36)
JDM^c^	9 (25)	2 (18)
Behcet’s/CAPS^d^/CRMO^e^/PFAPA^f^	5 (14)	4 (36)
Castleman/mixed connective tissue/morphea/rare genetic disorder/scleroderma/SLE^g^	8 (22)	1 (9)

a Parents and children and young people may not be from the same families.

b JIA: juvenile idiopathic arthritis.

c JDM: juvenile dermatomyositis.

d CAPS: cryopyrin-associated periodic syndrome.

e CRMO: chronic recurrent multifocal osteomyelitis.

f PFAPA: periodic fever aphthous stomatitis.

g SLE: systemic lupus erythematosus.

#### Descriptive Statistics

A total of 26 (70%) parents stated that they had heard of ChatGPT or other GenAI before, but only 14 (39%) of these said they had used any AI platforms. Of those who had, they used it for work purposes (n=11), while others (n=3) used it for general questions or “only experimented” with it. On the other hand, all 11 children and young people (100%) had heard of the technology. Less than half (n=5, 45%) had used it before; for learning (n=3), research work (n=1), and asking general questions “like the Snapchat AI” (n=1). Participants rated their self-confidence in using ChatGPT or similar GenAI technology on a 5-point Likert scale (1=not at all confident, 5=extremely confident), which was found to be low for both parents (median 2, IQR 1-3) and children and young people (median 2, IQR 1-4).

Participants were also asked to rate their likelihood of using ChatGPT or similar GenAI technology on a 5-point Likert scale (1=not at all likely, 5=extremely likely). Children and young people gave a high rating for their likelihood to use a specific chatbot (median 4, IQR 3-4) compared to ChatGPT (median 2, IQR 1-4), with a larger discrepancy indicated in the latter. Parents also rated the intended use of a specific chatbot (median 4, IQR 4-5) higher than ChatGPT (median 3, IQR 2-4) in managing their child’s condition. Additionally, parents’ ratings for using ChatGPT for their child’s health were slightly lower than using it for their own health (median 4, IQR 2-4).

#### Inferential Statistics

A Wilcoxon signed rank test confirmed that there was a statistically significant difference in parents’ attitudes toward using either a general or specific chatbot to assist in managing their child’s health (*z* score=3.8; *P*<.001; *r*=0.63). Children and young people’s attitudes were consistent with those of their parents (*z* score=2.5; *P*=.006; *r*=0.75). A total of 21 parents and 8 children and young people self-reported to be more likely to use a specific health chatbot than a general chatbot like ChatGPT. The likelihood of use was tied among 13 parents and 2 children and young people. In addition, 2 parents were more inclined to use ChatGPT than a specific chatbot for their child.

#### Qualitative Analysis of Questionnaire

Two follow-up questions were asked to better understand the reasons behind their ratings. Three themes were identified: accessibility, trust, and credibility in a chatbot, and specificity of chatbot design. Each theme was divided into subthemes, with quotes from participants as supporting evidence. Two subthemes in accessibility and trust and credibility did not include any quotes from children and young people because their responses were not relevant to the particular subthemes.

### Theme 1: Accessibility

#### Limited Accessibility to Health Care Professionals

None of the children and young people mentioned the role of health professionals, whilst one participant noted their dependence on their parents. Parents expressed how using a chatbot can mitigate the issue of having limited accessibility to health care professionals when they have questions about their child’s health. As a result, many noted that they seek advice anywhere and appreciate any help. For example:

*Any help is better than no help*.

[P2]

*I know the doctors do not have the time to spend with us to answer all our questions*.

[P22]

#### Efficient Tool for Information-Seeking

One child or young person mentioned that they did not think of using ChatGPT for seeking health information before but for academic purposes only. Parents perceived the chatbot to be an efficient tool in terms of time and accessibility for information seeking, even when they were specifically asked about ChatGPT.

*I think it’s the way forward to get immediate access to health information, I’ve just been hesitant to use it previously but can now see the benefits of using it*.

[P9]

#### Comprehension Accessibility

Another aspect of accessibility was the level of comprehension of health information. Parents thought chatbot-generated information could be rational, tailored, and understandable for the child. A parent said they would value tailored chatbot answers due to challenges with accessing health professionals.

*Because it would give rational and factual information rather than trying to get answers that would not be appropriate for my child*.

[P14]

### Theme 2: Trust and Credibility in a Chatbot

#### Concerns Regarding ChatGPT’s Inaccuracy

Both parents and children and young people expressed a lack of trust and confidence regarding the inaccuracy of ChatGPT on 2 levels: its open-source nature and its processing ability of complex medical information. Participants thought that ChatGPT could learn from sources that are not verified, resulting in unreliable, fabricated, or biased output. Participants also expressed distrust toward ChatGPT specifically for health care purposes, due to how complex medical information could be.

*[ChatGPT] It is open source so [it] may use unreliable or biased sources and it is dependent upon the prompts provided so in a medical context it could be easy to get it wrong and receive inaccurate responses*.

[P34]

*Mainly [I have not used it] because I hadn’t thought of doing so. After some thought, I wouldn’t trust the information to be accurate*.

[CYP3]

#### Unfamiliarity With Using the Technology Limits Trust

Some parents voiced their unfamiliarity with chatbot technology which led them to not having tried ChatGPT before. Therefore, they were not certain about its usefulness. Yet, all parents seemed open-minded to give the technology a try.

*I prefer to speak to a person who can understand nuance but would be open-minded to give AI a go ... the proof would be in the experience*.

[P32]

#### Reliance on Other Credible Sources

Participants compared chatbot use for health care to other sources that are deemed more credible, such as NHS resources and published research papers. Some participants sounded hesitant as they said that they expressed uncertainty about ChatGPT’s accuracy and reliability.

*I tend to look for the NHS website or research papers that have been published on the topic rather than asking a chatbot type for answers more so because I then can cite the information and figure out where it’s all come from. I am not sure if you can do that with ChatGPT*.

[CPY4]

### Theme 3: Specificity of Chatbot Design

#### Active Involvement of Health Care Professionals

Input by health professionals and experts in the design of a specific chronic health-focused chatbot was perceived to increase its accuracy and reliability, compared to ChatGPT. Participants expressed increased comfort with using a specifically designed chatbot. However, participants’ perception was solely based on their expectations rather than experience as the chatbot has not been developed yet.

*Due to it being more regulated and designed by health professionals who will hopefully monitor effectiveness and reliability of content*.

[P25]

*If the information is given by a specialist who deals in these illnesses day in [and] day out, I am way more inclined to trust the information given due to it being a reputable source. It’s a confident way to ask information and receive in a familiar way with the added comfort of knowing it is from a reputable source. Which is why my opinion changed rather drastically in a positive way [compared to ChatGPT]*.

[CYP4]

#### Fit-for-Purpose

All participants expressed that they were more inclined to use a chatbot that is specialized in rheumatology compared to a general chatbot like ChatGPT, GenAI, or Google. The importance of specialization due to the rarity and complexity of conditions and symptoms was highlighted.

*It’s important to us to have a resource available for this chronic condition that is not the same as Google*.

[P28]

*If it’s specific to JDM, I would be far more likely to use it [than ChatGPT]. JDM is so rare, that questions wouldn’t be answered unless it was programmed specifically for JDM*.

[P5]

*Rheumatological conditions can have such a vast array of symptoms that a nonhealth backed system could become quite scaremongering in leading to false information. If using a platform that has already narrowed down the results to trusted rheumatology information, I would feel a lot more comfortable using it [than ChatGPT]*.

[CYP3]

## Discussion

### Principal Findings

Using a mixed methods approach, we were interested in exploring both parental and children and young people’s attitudes and behavioral intentions toward using ChatGPT, a general chatbot that is commercially available in a health care context (RQ1), as well as their attitudes and behavioral intentions toward using a chatbot that would be specifically designed for pediatric rheumatology. The major difference between these 2 applications concerns the content returned to families, with a specifically created disease-specific chatbot only having access to a “closed system” of physician-endorsed data, rather than the ability to search the internet and bring back results with no safety netting from a health professional. We wanted to investigate whether the behavior intentions differed and if so, how (RQ2). Opportunities and concerns raised by children and young people and parents toward chatbot features and content were identified to indicate practical design implications (RQ3).

### Children and Young People and Parental Attitudes and Behavioral Intentions Toward ChatGPT and a Specific Chatbot (RQ1, RQ2)

The themes that emerged from the qualitative data contribute to our understanding of participants’ attitudes and behavioral intentions supported by the quantitative data. The accessibility of the chatbot to families was a key influencing factor in adoption intention. Parents explained that they would be likely to use the chatbot as they saw it as a time-efficient and easily accessible tool to turn to when health professionals are not available. Parents found chatbot technology, including ChatGPT, to be more direct and quicker in giving them the answers they need than other web-based resources. This may explain why parents were equally likely to use it for their own health and their child’s health. While it was not measured whether parents have health conditions themselves, they seemed to hold some health behavior motivation and curiosity toward a new accessible intervention. The findings are in line with previous research, which identified curiosity, perceived ease-of-use, and convenience to be motivators of health care chatbot adoption [21,42]. Chatbots, whether generally or specifically designed, could bring convenience to parents when health information-seeking. The integration of GenAI into the health care system is believed to have a positive impact on supporting medical research and decision-making for patients, families, and health care workers [43].

Our results showed that there was a statistically significant difference between participants’ likelihood of using ChatGPT and a specific chatbot for managing their child’s chronic health condition. Both parents and children and young people were significantly more likely to use the specific chatbot than ChatGPT for this purpose.

Trust and credibility in a chatbot was another theme identified. It highlighted participants’ concerns regarding ChatGPT’s accuracy which limited trust. They questioned its reliability due to open-sourcing and fabrication tendencies. Existing studies have consistently identified trust as a crucial factor in converting new users to adopt a novel technology intervention like a chatbot across contexts [21-23]. Additionally, participants thought that ChatGPT might not understand complex medical information in pediatric rheumatology and provide accurate responses. Participants’ unfamiliarity with ChatGPT might have also contributed to the overall low rate of current use. Despite finding ChatGPT accessible, participants would rather obtain internet resources from trusted sources, like the NHS website. Participants demonstrated careful evaluation of ChatGPT content, which is congruent with how patients processed and sought health information on the internet in Sillence et al [44]. While perceived ease-of-use and convenience could increase adoption intention to using a health care chatbot, users must first establish trust [23]. This is supported by Abbasian et al [45] who found that accuracy, trustworthiness, and empathy should be assessed when evaluating health care–specific chatbots.

What differentiated participants’ attitudes toward ChatGPT and the specific chatbot was the addition of health professionals’ input and the specific design focus in the latter. The theme of specificity of chatbot designs showed that users expected the specific chatbot to be specially trained to recognize information related to rheumatological conditions. A strong need for a resource specific to pediatric rheumatology was expressed due to the disease’s complexity. The specialized tool was perceived to be more reliable than ChatGPT, increasing users’ trust in its evidence-based input and monitored output. Participants displayed a high level of trust toward the specific chatbot and made comparisons to ChatGPT. Participants’ trust toward the specific chatbot might be explained by the theory of planned behavior [20]. It posits that subjective norm influences behavioral intention, besides attitudes. Participants might have attributed their trust in health providers to trust in the chatbot [23,46]. Participants of this study have demonstrated great engagement with the wider research project, which is advocated by health professionals they trust. Hence, families might have relied on health professionals’ opinions to form a strong subjective norm to follow, thereby increasing their adoption intention. This is further supported by findings from the review by Tangsrivimol et al [47] assessing the benefits and limitations of currently available chatbots such as ChatGPT and similar chatbots relying on GenAI. They have highlighted issues with existing chatbots by discussing ethical considerations and challenges with data governance and privacy concerns, as well as issues relating to inaccurate and misleading information provided by freely available chatbots based on erroneous algorithms and hallucination. Using an LLM for managing a child’s illness raises ethical concerns around safety, accountability, and privacy. Incorrect advice could cause harm, and there is no clear responsibility if things go wrong. Sharing sensitive health information also risks breaching confidentiality. Additionally, reliance on LLMs might undermine trust in health care professionals and could introduce bias, leading to unequal care. The aim of health care–specific chatbots is to overcome these issues by providing secure platforms and providing content, and features tailored to the audiences they are targeting, whilst still encouraging engagement with existing health care teams.

Besides trust, another concern raised in previous literature on health care chatbot adoption was the lack of humanness, compared to interactions with a health professional [21]. However, we argue that lack of humanness may not always be a concern. Reducing human-human interactions could be beneficial, such as creating a stigma-free safe space. Under the theme of accessibility, the subtheme of comprehension accessibility discussed the need for a tool that can provide understandable, tailored, and rational information. Indeed, stigmatizing health-related experiences could reduce patients’ engagement in medical care, treatment adherence, and help-seeking behavior [48]. Therefore, chatbot use might reduce any perceived stigma in patient-clinician interactions and provide tailored answers that users can understand. As a result, it could promote positive patient-clinician relationships and treatment outcomes.

Through analyzing families’ attitudes and behavioral intentions, it was identified that users need a tool that is highly accessible to compensate for limited contact with health professionals; that they can trust, such as being backed by credible experts; and that they can depend upon to provide accurate answers to questions specific to pediatric rheumatology. However, it is important to note that this tool is seen as an aide to support the family in everyday life situations, and should be a trusted source of information. Moreover, it should not be used as a replacement for existing care provided by health care teams but rather as a platform that should be used in conjunction with existing face-to-face care from health care providers.

### Opportunity of a Pediatric Chatbot (RQ3)

Through concept testing, the visual imagery and storytelling narrative used by ChatGPT were well-liked and thought to be appropriate for children and young people. However, they noted that children of the same age could behave and think very differently. In line with Vygotsky’s [49] sociocultural theory, children’s cognitive development is influenced by their social interactions and environment, irrespective of biological age. Hence, it might not be effective to dictate the level of comprehension in chatbot-generated output solely by age.

Younger participants and adults expressed different preferences for chatbot features and tone. Currently, parents are identified as the main user who will primarily use the chatbot either by themselves or with their children. Participants in concept testing discussed that children and young people should be considered as its own user group. There is therefore an opportunity to create a separate user interface and persona to engage children.

A study examined the impact of personas in a health care chatbot on adult users’ engagement [50]. It drew on the social response theory [51], which argues that users react to computers with human-like attributes similarly to human-human interactions. The study created the personas representing a hospital institution, expert, peer, and dialogical self to measure the effect of age and gender on affective bond and use. While the study concluded with mixed findings, future research could investigate the impact of different chatbot personas on intervention engagement in children, young people, and parents. However, the functionality and usefulness of a chatbot intervention should still be prioritized over social features [52]. This is particularly highlighted by a scoping review that reviewed health-related chatbots more closely. They found that mental health and well-being–promoting chatbots need to prioritize content and functionality above everything else. For example, they found that only 44% of chatbots successfully addressed suicidal thoughts. Thus, demonstrating the need to focus on safety to safeguard vulnerable populations [53].

### Limitations

Since participants were recruited voluntarily, they may be subject to social desirability bias toward the research project and team, as well as the recruitment of families from social media who equally may be biased toward AI. Additionally, some participants may have completed both the workshop and questionnaire, potentially increasing bias compared to those who only completed the survey. However, by asking participants to explain their ratings on chatbot use, they were encouraged to engage more deeply with the topic which increased objectivity [52].

The forced-choice design limits interpretation, as participants had to select the preferred option even if they were dissatisfied with both. This approach captures relative preference but may obscure low overall acceptance. Participants’ attitudes were also measured based on a hypothetical scenario of chatbot use, which may not be representative of a real-life situation. However, a study found that behavior is better predicted by actual experience, than attitudes that were formed based on the stimuli provided by the researcher [54]. It was not feasible in this study since the chatbot is yet to be developed. Future research could compare changes in participants’ attitudes after the chatbot has been developed. This could generate insights into how well the chatbot is embedded in users’ everyday lives, which is a common concern in AI health care technology [18].

While the use of a mixed methods approach has mitigated some limitations, it increased the complexity of the study. Questions from the questionnaire measure generated both quantitative and qualitative data. This was in addition to the qualitative data from the family workshop, which consisted of primary and secondary datasets. In addition, findings from one method slightly overlapped with findings from another. As a result, it increased the difficulty in data processing and interpretation. Findings from different methods had to be carefully evaluated to ensure a holistic understanding of the research problem and research questions and a clear audit trail and regular team meetings were essential to identify potential biases. Additionally, the sample size for children and young people was too small to allow for more in-depth interpretations of the findings. Children and young people were included in this study as they attended the workshop alongside their parents, and it provided an opportunity to collect preliminary data on their views. Further research could investigate their viewpoint in more detail to understand their views on GenAI and health-specific chatbots.

As health care chatbots can have a wide spectrum of applications and target audiences, a limitation in the research field is that it is hard to compare study results. To illustrate, a recent systematic review on health care chatbots by Laymouna et al [55] found that only 10 out of 161 (6%) studies reviewed were designed for chronic health patients. Health care chatbots could also serve different functionalities, such as everyday management, education, and administrative support. Another review on chatbots for chronic patients [56] only discovered 10 published studies, confirming that the research field is still in its infancy. Moreover, 60% of studies looked at different chronic health conditions. It is important therefore to consider developing specialized chatbots for different conditions as they potentially vary in symptoms, causes, and management.

### Conclusions

This study contributes to the body of literature on AI in pediatric health care, by exploring parental and children and young people’s attitudes and acceptance toward a specialized GenAI chatbot for pediatric rheumatology. While this study focused on pediatric rheumatology, findings might be generalizable to other chronic pediatric health conditions. The study also adds to the existing studies on health care chatbots, as the preference for a specific chatbot is demonstrated over a general chatbot, like ChatGPT. Families wanted a chatbot that was accessible and able to “translate” medical information into language suitable for individuals’ comprehension levels. Moreover, trust and credibility regarding the chatbot’s input, design, and regulation promoted users’ adoption intention. As the current “IMPACT” study is based on the concept of a specialized pediatric chatbot, future work should continue to involve users in chatbot development. A chatbot developed for specific pediatric conditions could drastically change the way patients and caregivers seek health information digitally. Not only will families receive the information they want when they need it but also they could be empowered in chronic health management, whilst maintaining a partnership with their child’s health professionals.

## Supplementary material

10.2196/70409 Multimedia Appendix 1 World café concept testing questions.

## Abbreviations

AI: artificial intelligence
GenAI: generative artificial intelligence
IMPACT: Interventions to Improve Mental Health Support in Families With Children and Young People With Chronic Rheumatological Conditions
LLM: large language model
NHS: National Health Service
REDCap: Research Electronic Data Capture
