# Supplementary material for: Exploring the Acceptance and Opportunities of Using a Specific Generative AI Chatbot to Assist Parents in Managing Pediatric Rheumatological Chronic Health Conditions: Mixed Methods Study
Source: JMIR Pediatr Parent. 2025 Jul 1;8:e70409. doi: 10.2196/70409 (PMC12236638; doi:10.2196/70409)
Supplement: Multimedia Appendix 1 [file pediatrics-v8-e70409-s001.docx]

**ChatGPT-generated output and questions**

| Q: Explain Juvenile Idiopathic Arthritis to a 6 year old  A: Alright, let's make it simple and easy to understand!  Imagine your body is like a big castle, and inside this castle, there are lots of rooms. Each room has its own special guards called "immune cells." These guards are there to protect the castle from any bad guys, like germs or viruses, that might try to sneak in and make you sick.  Sometimes, in some kids, these guards get a little confused. Instead of just protecting the castle, they start to attack the castle itself, especially the joints – these are like the doors and hinges in the castle. When this happens, it makes the joints swollen, stiff, and sometimes, it can even hurt a bit.  So, Juvenile Idiopathic Arthritis is when these guards in some kids get mixed up and start attacking the body's own joints, causing them to feel sore and not work as well as they should. But don't worry, doctors have special ways to help calm down these confused guards and make the joints feel better again, so you can go back to playing and having fun! |
| --- |

| Q: Explain Juvenile Idiopathic Arthritis to a 13 year old  A: Alright, let's break it down in a way that's easy to understand:  Imagine your body is like a big house with lots of rooms. Each room represents a different part of your body, like your muscles, bones, and organs. Now, sometimes in some kids, there's a problem in one of these rooms, specifically in the joints, which are like the hinges that help your body move smoothly, like the doors in a house.  When we say "Juvenile Idiopathic Arthritis" (JIA), it's like saying there's something wrong with those hinges in some kids, and we're not exactly sure why it's happening. "Arthritis" means there's inflammation or swelling in the joints, which can make them hurt, feel stiff, or hard to move.  Now, "Juvenile" just means it happens in kids, like you, who are younger than adults. "Idiopathic" is a fancy word that means doctors don't know the exact cause for sure. So, it's like a mystery that doctors are trying to solve!  Just like how each house is different, JIA can affect kids in different ways. Some might have just a few joints that are sore, while others might have more. But the good news is, doctors and specialists called pediatric rheumatologists know a lot about these problems and can help figure out the best ways to make those sore joints feel better, so you can get back to doing all the fun things you love! |
| --- |

| Q: explain Juvenile Dermatomyositis to a 10 year old  A: Alright, imagine your body is like a castle, and it has knights inside to protect it from bad things. Now, sometimes, in some kids, the knights get a bit confused and start attacking the castle instead of protecting it.  Juvenile dermatomyositis is a big word that means sometimes the knights in your body, which are your immune system, get mixed up and start attacking your muscles and skin by mistake. This can make your muscles feel weak and sore, and sometimes your skin might get red or itchy.  But just like in a real castle, there are ways to help calm down those confused knights and make them behave properly again. Doctors have special tools (medicines and treatments) to help your body fight back against those naughty knights, so you can feel better and go back to playing and having fun like you used to. It might take some time, but with the right help, your body will get back to its strong and healthy self.  Q: Can you make it a bit more technical?  A: Sure, let's add a bit more detail.  Imagine your body as a kingdom, with different parts like the muscles and skin being the castles and walls that protect it. Now, in some kids, there's a condition called Juvenile Dermatomyositis (JDM). In this condition, there's a problem with the immune system, which is like the army of the kingdom. Instead of just protecting the kingdom, the immune system gets confused and starts attacking the muscles and skin.  Dermatomyositis means "inflammation of the skin and muscles." So, "juvenile" means it happens in kids. When the immune system attacks the muscles, it can make them weak and sore. That's why you might feel tired or have trouble moving sometimes. And when it attacks the skin, it can cause redness, rashes, or itchiness.  Doctors use special tools, like medicines and treatments, to help calm down the overactive immune system. These tools are like strategies to make the army stop attacking the wrong things and focus on protecting the kingdom again. With the right help from doctors and treatments, the body can fight back and get stronger, just like a kingdom that defends itself against invaders. It may take some time, but eventually, your body will get back to feeling healthy and strong.  Q: Why has it occured?  A: Juvenile dermatomyositis (JDM) occurs when the immune system, which normally protects the body from harmful invaders like viruses and bacteria, mistakenly attacks its own tissues. While the exact cause is not fully understood, it's believed to be a combination of genetic factors and environmental triggers.  Genetic predisposition plays a role, as certain genes may make some children more susceptible to developing JDM. However, not everyone with these genetic factors will develop the condition.  Environmental factors, such as infections or exposure to certain substances, may trigger the immune system to start attacking the body's own tissues in susceptible individuals. However, these triggers can vary from person to person, and not everyone exposed to them will develop JDM.  Overall, JDM is thought to result from a complex interplay between genetic susceptibility and environmental triggers, leading to the immune system dysfunction seen in the condition. |
| --- |

Read the answers created by computer (ChatGPT) and let’s discuss:

1. What are your general thoughts on the answers generated by the computer?
2. How accurate do you think the computer-generated responses are?
3. How appropriate do you think the computer-generated responses are, targeting children of 6-year-old and 13-year-old respectively?
4. How different do you think the computer-generated responses are compared to responses provided by a doctor?
5. Do you have any criticism regarding the computer-generated responses?
